# Species and genotypic diversity of non-tuberculous mycobacteria isolated from children investigated for pulmonary tuberculosis in rural Uganda

**DOI:** 10.1186/1471-2334-13-88

**Published:** 2013-02-18

**Authors:** Benon B Asiimwe, Godwins B Bagyenzi, Willy Ssengooba, Francis Mumbowa, Gerald Mboowa, Anne Wajja, Harriet Mayanja-Kiiza, Philippa M Musoke, Eric Wobudeya, Gunilla Kallenius, Moses L Joloba

**Affiliations:** 1 Department of Medical Microbiology, Makerere University College of Health Sciences, P.O. Box 7072, Kampala, Uganda; 2 Department of Clinical Science and Education, Karolinska Institutet, Södersjukhuset, Research Center, SE-118 83, Stockholm, Sweden; 3 Infectious Diseases Institute, Makerere University College of Health Sciences, P.O. Box 7072, Kampala, Uganda; 4 Department of Paediatrics and Child Health, Makerere University College of Health Sciences, P.O. Box 7072, Kampala, Uganda

## Abstract

**Background:**

Smear microscopy, a mainstay of tuberculosis (TB) diagnosis in developing countries, cannot differentiate *M. tuberculosis* complex from NTM infection, while pulmonary TB shares clinical signs with NTM disease, causing clinical and diagnostic dilemmas. This study used molecular assays to identify species and assess genotypic diversity of non-tuberculous mycobacteria (NTM) isolates from children investigated for pulmonary tuberculosis at a demographic surveillance site in rural eastern Uganda.

**Methods:**

Children were investigated for pulmonary tuberculosis as part of a TB vaccine surveillance program (2009–2011). Two cohorts of 2500 BCG vaccinated infants and 7000 adolescents (12–18 years) were recruited and followed up for one to two years to determine incidence of tuberculosis. Induced sputum and gastric aspirates were processed by the standard *N*-acetyl *L*-cysteine (NALC)-NaOH method. Sediments were cultured in the automated MGIT (Becton Dickson) liquid culture system and incubated at 37°C for at least six weeks. Capilia TB assay was used to classify mycobacteria into MTC and NTM. The GenoType CM/AS assays were performed to identify species while Enterobacterial Repetitive Intergenic Consensus (ERIC) PCR genotyping was used to assess genetic diversity of the strains within each species.

**Results:**

Among 2859 infants and 2988 adolescents screened, the numbers of TB suspects were 710 and 1490 infants and adolescents respectively. The prevalence of NTM in infant suspects was 3.7% (26/710) (95% CI 2.5–5.2) while that in adolescent suspects was 4.6% (69/1490) (95% CI 3.6–5.8). On culture, 127 isolates were obtained, 103 of which were confirmed as mycobacteria comprising of 95 NTM and eight *M. tuberculosis* complex. The Genotype CM/AS assay identified 63 of the 95 NTM isolates while 32 remained un-identified. The identified NTM species were *M. fortuitum* (40 isolates, 63.5%), *M. szulgai* (9 isolates, 14.3%), *M. gordonae* (6 isolates, 9.5%), *M. intracellulare* (3 isolates, 4.7%), *M. scrofulaceum* (2 isolates, 3.2%), *M. lentiflavum* (2 isolates, 3.2%), and *M. peregrinum* (1 isolate, 1.6%). Genotyping did not reveal any clustering in *M. intracellulare*, *M. gordonae* and *M. szulgai* species. *M. fortuitum*, on the other hand, had two clusters, one with three isolates of *M. fortuitum* 1 and the other with two isolates of *M. fortuitum* 2 subspecies. The remaining 35 of the 40 isolates of *M. fortuitum* had unique fingerprint patterns.

**Conclusion:**

*M. fortuitum* is the most common cause of infection by NTM among Infants and adolescents in rural Uganda. There is a varied number of species and genotypes, with minimal clustering within species, suggesting ubiquitous sources of infection to individuals in this community.

## Background

The non-tuberculous mycobacteria (NTM) include those *Mycobacterium* species that are not members of the *Mycobacterium tuberculosis* complex, the causative agent of pulmonary tuberculosis (TB), and NTM diseases share clinical signs with TB, causing a clinical dilemma with regard to therapy for patients [1]. In parts of the world where infection by acid-fast bacilli could be due to either *M. tuberculosis* or NTM and because of the need to institute appropriate public health and chemotherapeutic measures immediately, the causative agent must be identified. However, in children, the diagnosis of pulmonary tuberculosis (PTB) is difficult since disease is often pauci-bacillary therefore the bacteriological confirmation, the gold standard, may not be adequate. In high disease-burden and resource-limited settings, clinicians rely on history-taking, tuberculin skin testing, and chest radiography for investigation of PTB in children [2], a practice that may not distinguish TB from NTM lung disease. A further danger lies in the fact that detection of non-tuberculous acid-fast bacilli in respiratory secretions by direct smear microscopy might be misinterpreted as PTB unless mycobacterial species identification is available [3]. In Uganda, there is limited information on NTM infection in children in spite of the fact that NTM have been isolated from the environment in rural communities [4] and domesticated animals [5,6]. Therefore, the prevalence, species and possible transmission routes of NTM infection in humans remain largely unknown.

DNA strip technology (line probe assays) based on the reverse hybridization of PCR products to their complementary probes has been used for the simultaneous detection and identification of mycobacteria. The GenoType Mycobacterium (Hain Lifescience GmbH, Nehren, Germany) is a commercial DNA strip assay used for the detection and identification to the species level of mycobacteria obtained from positive liquid or solid cultures. In Uganda, these assays have been successfully used on both clinical [7,8] and veterinary [5] isolates. For the NTM, the GenoType assay comprises two kits: the GenoType CM (for common mycobacteria) and GenoType AS (for additional species) assays, providing probes for 14 and 16 species, respectively.

In an investigation of *Mycobacterium fortuitum* group of strains causing post-mammoplasty infections in Campinas, Brazil, using four molecular typing methods [pulsed-field gel electrophoresis (PFGE) and three PCR-based techniques: 16S-23S rRNA internal transcribed spacer (ITS) genotyping; randomly amplified polymorphic DNA (RAPD) PCR; and enterobacterial repetitive intergenic consensus (ERIC) PCR], PFGE produced the most discriminatory patterns, but was technically difficult, labour-intensive, and required expensive equipment [9]. PCR-based techniques, on the other hand, were less expensive, easier to perform, generated results in a timely fashion, and required only well-standardized protocols and equipment available in most laboratories. More interestingly, ERIC-PCR generated profiles that formed the same clonal groups as those recognized by the gold standard PFGE, and it was sufficiently discriminative to be useful for the generation of reliable and timely results in the investigation of outbreaks caused by *M. fortuitum*[9]. Our study characterized NTM isolates from children investigated for PTB in a demographic surveillance site in rural Uganda, using the GenoType CM and GenoType AS to identify the species, and ERIC-PCR to assess genetic diversity of the strains within each species.

## Methods

### Study design

Two cohorts of BCG vaccinated infants and adolescents (12–18 years) were recruited and followed up for one 1–1.5 years to determine incidence of TB. Cohorts were recruited from a community in the Iganga/Mayuge Demographic surveillance Site, a largely rural setting in Eastern Uganda. Suspects, identified through active case finding and investigated for TB, were defined as any one of the following: presence of symptoms suggestive of TB for more than 2 weeks, history of household TB contact, and a positive tuberculin skin test(>10 mm or >5 mm in HIV positive). Four samples were collected from infants (2 induced sputum and 2 gastric aspirates) and two from adolescents (1 early morning sample and 1 coached sputum sample). Samples were transported, on the day of collection, to the BSL3 TB laboratory in the Department of Medical Microbiology, Makerere University College of Health Sciences.

### Culture and isolation

Samples were processed by the standard *N*-acetyl *L*-cysteine (NALC)-NaOH method [10]. Specimens (2.5 to 10 ml) were processed using 1% NaOH/NALC method and concentrated at 4000 *× g* for 15 minutes. The sediment was reconstituted to 2.5 ml with phosphate buffer pH 6.8, to make the inoculum for the fluorescent smear microscopy, and cultures in the automated MGIT (Becton Dickson) liquid culture system were incubated at 37°C for at least six weeks. Only one culture isolated per study subject was considered for further analysis.

### Species identification

#### GenoType assay on isolates

Capilia TB assay, which has recently been evaluated for use in our setting [11], was used to classify the mycobacteria into MTC and NTM. The GenoType CM/AS (Hain Lifescience GmbH, Nehren, Germany) assays were performed on heat thermo-lysates according to the manufacturer’s instructions, using the reagents provided with the kits. GenoType CM was used to identify the common NTM, while any isolate not identified by this assay was tested with the GenoType AS assay, which provides probes for a series of additional NTM. The isolated MTC were identified to species level by region of difference (RD) analysis as described elsewhere [12].

### Enterobacterial repetitive intergenic consensus (ERIC) PCR genotyping

ERIC-PCR was performed with primers ERIC-1R (5′-AT GTAAGCTCCTGGGGATTCAC-3′) and ERIC2 (5′-AA GTAAGTGACTGGGGTGAGCG-3′) [9], in 10 μl reactions containing 25 pmol of each of the two primers, 10× Custom PCR master mix (Thermo scientific ABgene, UK), Nuclease free water (Quiagen, Germany), 1 U of Taq DNA polymerase, 2 μl of heat thermo-lysate template in a PTC Thermocycler using the following conditions: initial denaturation at 95°C for 5min; 30 cycles at 90°C of denaturation for 30 sec, annealing at 40°C for 1 min, extension at 72°C for 2 min and final extension at 72°C for 10 min and products held at 4°C until run on a gel. The products were separated by electrophoresis using a 2% w/v agarose gel (agarose electrophoresis grade; Fisher scientific) using 1× TBE buffer at a voltage of 120 for 3 hours. A mixture of λ DNA/HindIII and ϕχ174 DNA/HaeIII fragments (Promega Corp, Madison, WI, USA) was used as the reference marker.

### Data analysis

The test strips were fixed on a data sheet. Development of conjugate, universal and genus control lines were assessed carefully. In accordance with the manufacturer's instructions, only bands whose intensities were as strong as or stronger than the universal control line were considered. ERIC-PCR gels were read on the camera and directly exported to BioNumerics software, version 5.0 (Applied Math’s, Kortrijk, Belgium) as fingerprint patterns. To determine the genetic relatedness of NTM strains in this study, dendrograms were constructed using the pair wise distance and the Jaccard index between fingerprint patterns by the un-weighted pair-group method of arithmetic averages (UPGMA) for the different species at a tolerance level of 2%. Proportions and 95% Confidence Intervals (CI) were determined by the on-line OpenEpi-epidemiological calculator (http://www.openepi.com/OE2.3/Menu/OpenEpiMenu.htm).

### Ethical issues

The study, a TB vaccine surveillance program, was approved by the Research and Ethics Committee of the School of Public Health of Makerere University, as well as the Uganda National Council for Science and Technology (UNCST). Parents/guardians were informed about the study and written informed consent was obtained from the parent or legal guardian.

## Results

### Prevalence of NTM

Among the infants and adolescents screened, the numbers of TB suspects were 710 and 1490 infants and adolescents respectively. The mean age for the adolescents was 14, ranging from 12 to 16 years, while the infants were aged between and four and 11 months. The prevalence of NTM in infant suspects was 26/710 (3.7%, 95% CI 2.5–5.2) while that in adolescent suspects was 69/1490 (4.6%, 95% CI 3.6–5.8). There was no smear positive NTM case in infants while only 14 were smear positive among the adolescents. Additionally, Capilia TB assay identified eight isolates, al being from adolescents, as belonging to the *M. tuberculosis* complex and these were later confirmed, by deletion analysis, to be *M. tuberculosis* strict sense with the characteristic deletion at the TbD1 locus and being intact for RD9.

### NTM species

In this study, 127 isolates were obtained on culture, 103 of which were confirmed as mycobacteria (95 NTM and eight *M. tuberculosis*) while the remaining 24 were other growth. The 95 NTM isolates available for analysis yielded 63 identifiable NTM by the Genotype CM/AS assay, comprising seven species, and 32 non-identifiable NTM. The most common NTM species identified were *M. fortuitum* (40 isolates, 63.5%), *M. szulgai* (9 isolates, 14.3%), and *M. gordonae* (6 isolates, 9.5%) (Table 1). Thirty of the 40 *M. fortuitum* isolates were from adolescents while in *M. szulgai* five of the nine were from adolescents. The other species identified in adolescents were four *M. gordonae*, two *M. intracellulare* and the only *M. peregrinum* identified in the study. All the two *M. lentiflavum* isolates identified were from infants. On the other hand, 25 of the 32 (78.1%) non-identifiable NTM isolates were from adolescents. Clearly, species distribution differed according to age group, with adolescents being more infected.

**Table 1:** Non-tuberculous mycobacterial isolates (n = 95) obtained from gastric lavage or induced sputum specimens in children investigated for pulmonary tuberculosis

**Isolates of non-tuberculous mycobacteria**	**No.**	**%**
**Identifiable NTM**	**n = 63**	**66.3**
*M. fortuitum*	n = 40	63.5
*M. szulgai*	n = 9	14.3
*M. gordonae*	n = 6	9.5
*M. intracellulare*	n = 3	4.8
*M. scrofulaceum*	n = 2	3.2
*M. lentiflavum*	n = 2	3.2
*M. peregrinum*	n = 1	1.6
**Non-identifiable NTM**	**n = 32**	**33.7**

*No.* number, % = percentage.

### NTM genotypes

**Figure 1:**
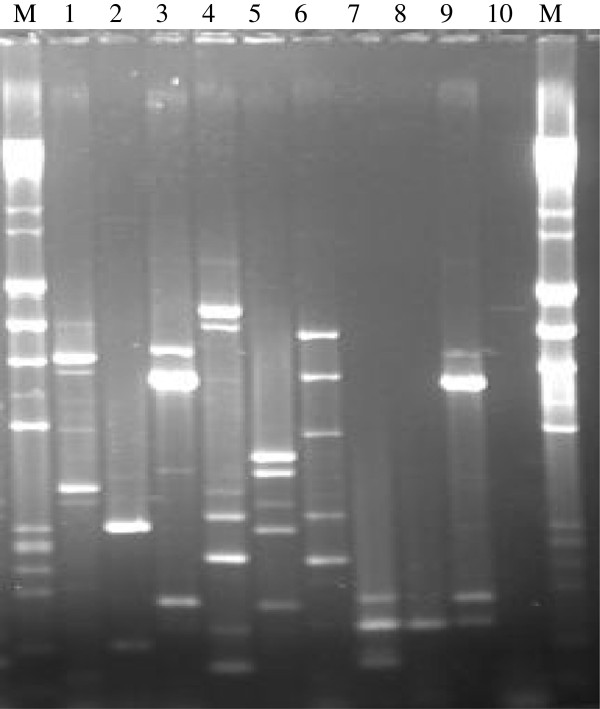
**A 2% agarose gel for ERIC PCR of 10 of the 103 isolates.** M = DNA ladder (marker), 1 to 10 shows the fingerprint of each isolate.

**Figure 2:**
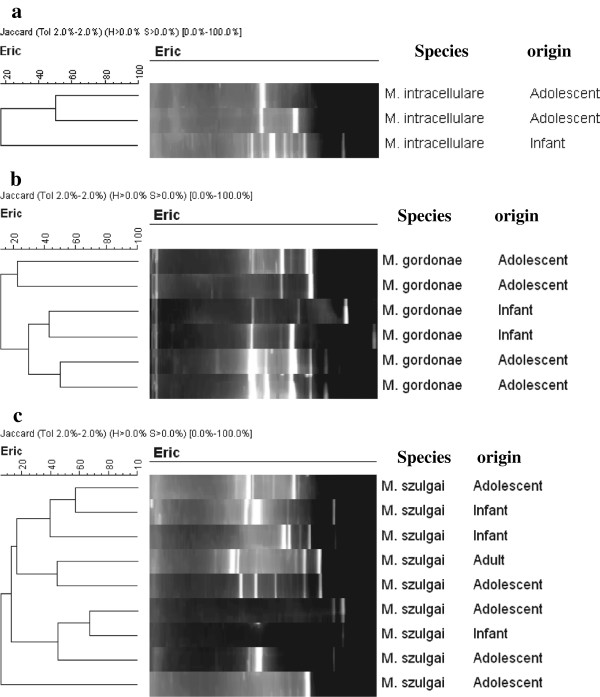
**ERIC-PCR profiles *for *a) *M. intracellulare, *b) *M. gordonae *and c) *M. szulgai *isolates analyzed by BioNumerics v.5.0.**

**Figure 3:**
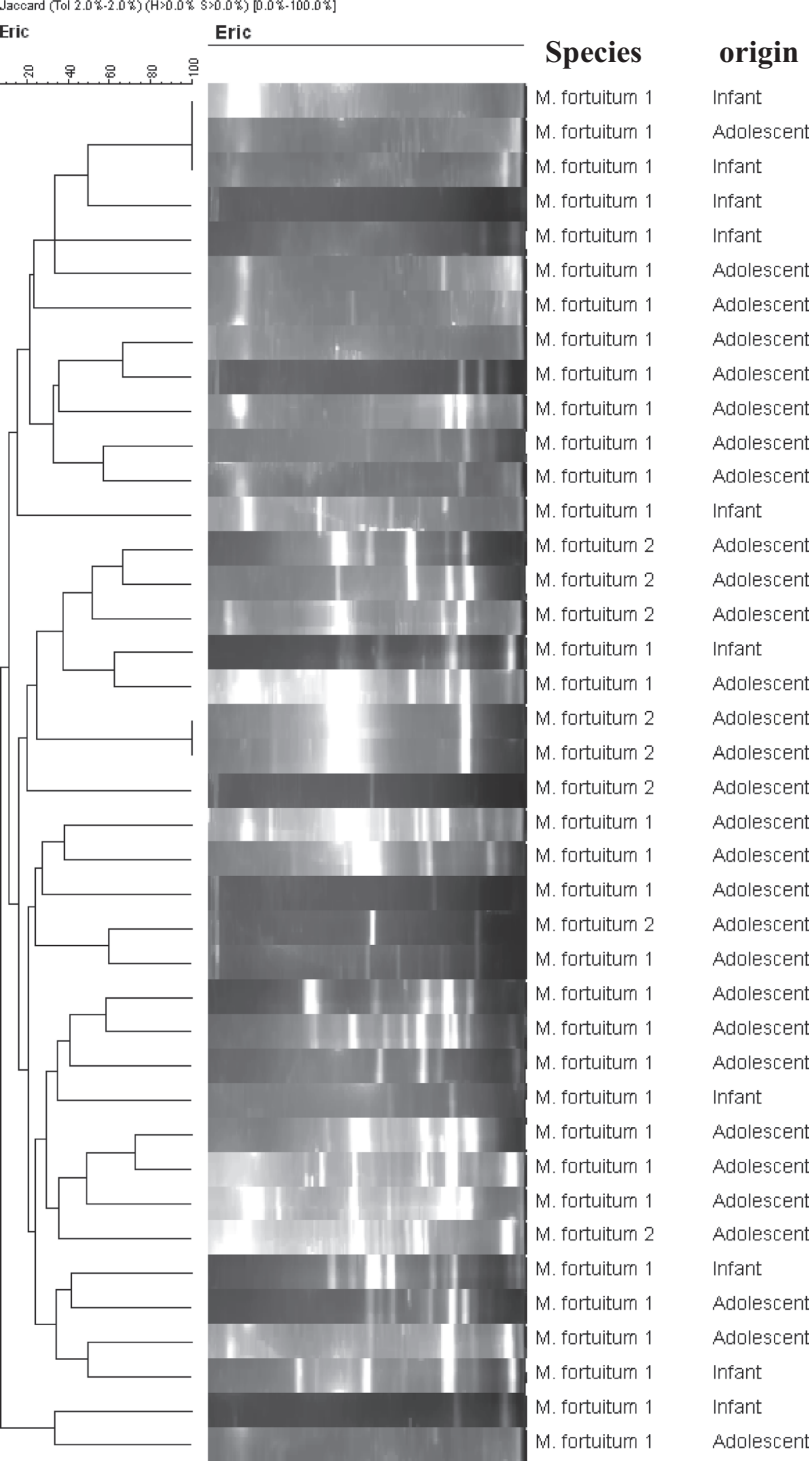
**ERIC-PCR profiles for *Mycobacterium fortuitum *isolates, analyzed by BioNumerics v.5.0.**

Only species with three or more isolates (*M. intracellulare*, *M. gordonae, M. szulgai* and *M. fortuitum*) were genotyped so as to understand the intra-species genetic diversity. Figure 1 shows a 2% agarose gel for ERIC-PCR of 10 isolates, which was later exported to the BioNumerics software for analysis. This technique was used to type all the 63 identifiable NTM isolates in the study. There was no clustering in *M. intracellulare*, *M. gordonae* and *M. szulgai* species (Figure 2a, b, and c respectively). However, there were two clusters among the 40 *M. fortuitum* isolates, one with three isolates (from two infants and one adolescent) of *M. fortuitum* 1 while the other had two isolates (from two adolescents) of *M. fortuitum* 2 subspecies (Figure 3). The remaining 35 of the 40 *M. fortuitum* isolates had unique fingerprint patterns.

## Discussion

The identification of NTM is important because positive microscopy, a main stay of TB diagnosis in developing countries, cannot differentiate *M. tuberculosis* complex from NTM infection, while pulmonary TB shares clinical signs with NTM disease, causing diagnostic and clinical dilemmas [2]. NTM isolated from humans can be found in ecosystems shared between humans and animals, and in soil and natural open water sources, all of which play a key role as sources of human infections [13]. Our study area is a rural agricultural setting, similar to other rural areas where studies have shown a high concentration of NTM in domesticated animals and the environment [4,14]. The current study has isolated and characterized NTM from respiratory secretions in more than 90 children, with a majority exhibiting symptoms suggestive of pulmonary TB.

We have identified seven species comprising of 65/95 (68%) of the NTM isolates in children from a rural Ugandan community. The most prevalent of the identified isolates, *M. fortuitum* (63.5%), is a ubiquitous contaminant and colonizer that can be isolated from environmental sources such as potable water systems and soil [15]. The other major species isolated were *M. szulgai* (14.3%), and *M. gordonae* (9.5%). In rural agro-pastoral Uganda, a recent study of 310 samples from soil, water and fecal matter from cattle and pigs isolated 48 NTM [4]. The major species identified in that study were 15 (31.2%) *M. avium* complex, 12 (25%) *M. fortuitum-peregrinum* complex, five (10.4%) *M. gordonae*, and five (10.4%) *M. nonchromogenicum*. Although there were no human samples analyzed in that study, the two sets of results clearly support an environmental link to the infection in the children in the current study. Our findings are in further agreement with those in other studies which observed that children in rural agricultural communities might be at greater risk of exposure to environmental NTM than their urban counterparts [16]. Furthermore, our study showed that there were more NTM isolated from adolescents (69/95) compared to infants (26/95), a finding in agreement with results from a similar study in South Africa [16], and this is thought to be due to increased environmental exposure in older children. While we isolated more *M. fortuitum*, the study in agro-pastoral ecosystems of Uganda showed that *M. avium intraceullare* complex was the predominant NTM isolated [4]. In our study 33.7% (32/95) of the isolates could not be identified; we thus recommend that new probes for the correct identification of more NTM species be sought.

Enterobacterial Repetitive Intergenic Consensus (ERIC) PCR was used to type all 63 identifiable NTM species in order to assess intra species clustering, hence commonality of sources of NTM infection in the study community. The nine *M. szulgai,* six *M. gordonae* and three *M. intracellualre* isolates did not cluster within the species, each isolate showing a distinct fingerprint pattern. Analysis of the 40 *M. fortuitum* isolates, on the other hand, showed unique fingerprint patterns in 35 isolates while the remaining five clustered into two, comprising of three and two isolates each. The cluster of three involved two infants and one adolescent sharing *M. fortuitum* 1 while the cluster of two involved adolescents with *M. fortuitum* 2 subspecies. However, the low fragment number of the isolates in the two clusters above is not sufficiently discriminative to evaluate these *M. fortuitum* clones. A similar study investigating *M. avium* mycobacterial lymphadenitis in children in The Netherlands using *IS*1245 Restriction Fragment length Polymorphisms (RFLP) analysis did not reveal any geographical clustering, with the 34 isolates in that study scattered over several clades [17]. The environment is the most likely reservoir for these infections, as there is no evidence of human-to- human or animal-to-human transmission, and the only aerosol transmission of NTM infection recorded to date was from shower water [18].

The exact main clinical relevance of these mycobacteria is that colonization may induce non-specific immune response and thereby leading to false positive reactions in the Mantoux test [19]. However, the infrequency of smear positivity relatively negates the concern for misdiagnosis of TB when using microscopy alone. Furthermore, if culture alone is used without genotypic identification, then *M. fortuitum* and other rapid growers are less likely to be confused for *M. tuberculosis*, thus the clinical implication may be less profound than previously stated.

## Conclusion

*M. fortuitum* is the most common cause of infection by NTM in this rural Ugandan community. There is a varied number of species and genotypes, with minimal clustering rate among the species, suggesting environmental sources of infection to individuals in this community.

## Competing interests

The authors declare that they have no competing interests.

## Authors’ contributions

BBA, PMM, HMK, GK, MLJ conceived the study, BBA, GB, WS, FM, GM performed the laboratory work, PMM, HMK, AW, EW, GK, MLJ supervised the work, BBA drafted the manuscript, PMM, AW, HMK, GK, MLJ critically revised the manuscript. All authors read and approved the final manuscript.

## Pre-publication history

The pre-publication history for this paper can be accessed here:

http://www.biomedcentral.com/1471-2334/13/88/prepub
